# Viable Cesarean Scar Ectopic Pregnancy Diagnosed by Ultrasound and Managed Surgically: A Case Report

**DOI:** 10.7759/cureus.90732

**Published:** 2025-08-22

**Authors:** Quang Dai La, Aiman Baloch, Muhammad Ayub, Sobia Ahmed, Farzana Jaffar, Rehana Sarwar, Francis Pryor, Pari Gul

**Affiliations:** 1 Biology, Texas A&M University, College Station, USA; 2 Medicine, The Innovative STEMagazine 501(c)3, College Station, USA; 3 Medicine, Mekran Medical College, Turbat, Turbat, PAK; 4 Radiology, Bolan Medical Complex Hospital, Quetta, PAK; 5 Medicine, Lake Erie College of Osteopathic Medicine, Erie, USA

**Keywords:** cesarean scar pregnancy, cesarean section, early pregnancy complication, ectopic pregnancy, gestational sac, obstetric imaging, surgical management, transabdominal ultrasound, uterine rupture, viable embryo

## Abstract

Cesarean scar ectopic pregnancy (CSEP) is a rare and potentially fatal form of ectopic gestation seen in a patient with a prior cesarean delivery, resulting in implantation of the fertilized ovum within the fibrous scar of the previous cesarean. We report a case of a 34-year-old female with a history of cesarean delivery who presented to the emergency department with complaints of lower abdominal pain and amenorrhea for nine weeks. Transabdominal ultrasound showed an empty uterine cavity and empty cervical canal, with a viable gestational sac implanted in the anterior lower uterine segment at the site of the previous cesarean scar. The patient underwent laparotomy with scar excision. Laparoscopy is not commonly utilized in our setting, as there is only one laparoscopic operating theater available, which is primarily used by general surgery; consequently, all gynecology and obstetrics procedures are performed through conventional open approaches. Surgical excision of the ectopic gestation was completed without intraoperative complications. This case reinforces the need for early recognition and surgical excision of viable CSEP in order to prevent disastrous complications like uterine rupture or massive hemorrhage. It also highlights the role that imaging plays in both diagnosis and surgical planning for patients who may want future reproductive opportunities.

## Introduction

Cesarean scar ectopic pregnancy (CSEP) is a rare and potentially life-threatening type of ectopic gestation where the embryo implants in the myometrial scar of a previous cesarean delivery [1]. The incidence of CSEP is increasing in parallel with the increasing global cesarean delivery rates and has a high risk of uterine rupture, massive hemorrhage, and hysterectomy if the diagnosis is delayed [1]. Early identification of CSEP is crucial, particularly through transabdominal and transvaginal ultrasound, and can help in determining management strategies and subsequent fertility preservation; suggestive imaging includes an empty uterine cavity, a gestational sac in the anterior lower uterine segment at the cesarean scar, and an absence of myometrium between the gestational sac and bladder [1,2].

CSEP was classified into subtypes using gestational sac location and myometrial thickness to better assess risk and formulate treatment options [2]. Management varies from systemic or intragestational methotrexate, surgical resection with or without scar repair, or more aggressive management depending on diagnosis, gestational age, and patient choice concerning their fertility [1,3].

To clarify, type II CSEP is defined by deep implantation of the gestational sac into the cesarean scar defect, infiltrating the uterine myometrium and bulging toward or through the serosal surface, and is therefore potentially associated with risk for early uterine rupture and bleeding [4]. A modern classification system proposed by Ban et al. further classified type II into IIa (gestational sac diameter ≤ 30 mm) and IIb (gestational sac diameter > 30 mm) based on mean sac diameter and anterior myometrial thickness (1-3 mm) for surgical individualized decision-making [5].

We report a case of a 34-year-old female with viable type II CSEP, managed surgically in a hemodynamically stable patient. The patient was able to complete the surgical excision of the ectopic gestation without intraoperative complications. This case underscores the role of early imaging and multidisciplinary planning in optimizing outcomes and preserving reproductive potential.

This case was presented as a poster at the Joint Conference of Body Imaging Radiological Society of Pakistan (BIRSP) and National Breast Radiological Society of Pakistan (NBRSP) on April 13th, 2025.

## Case presentation

**Figure 1:**
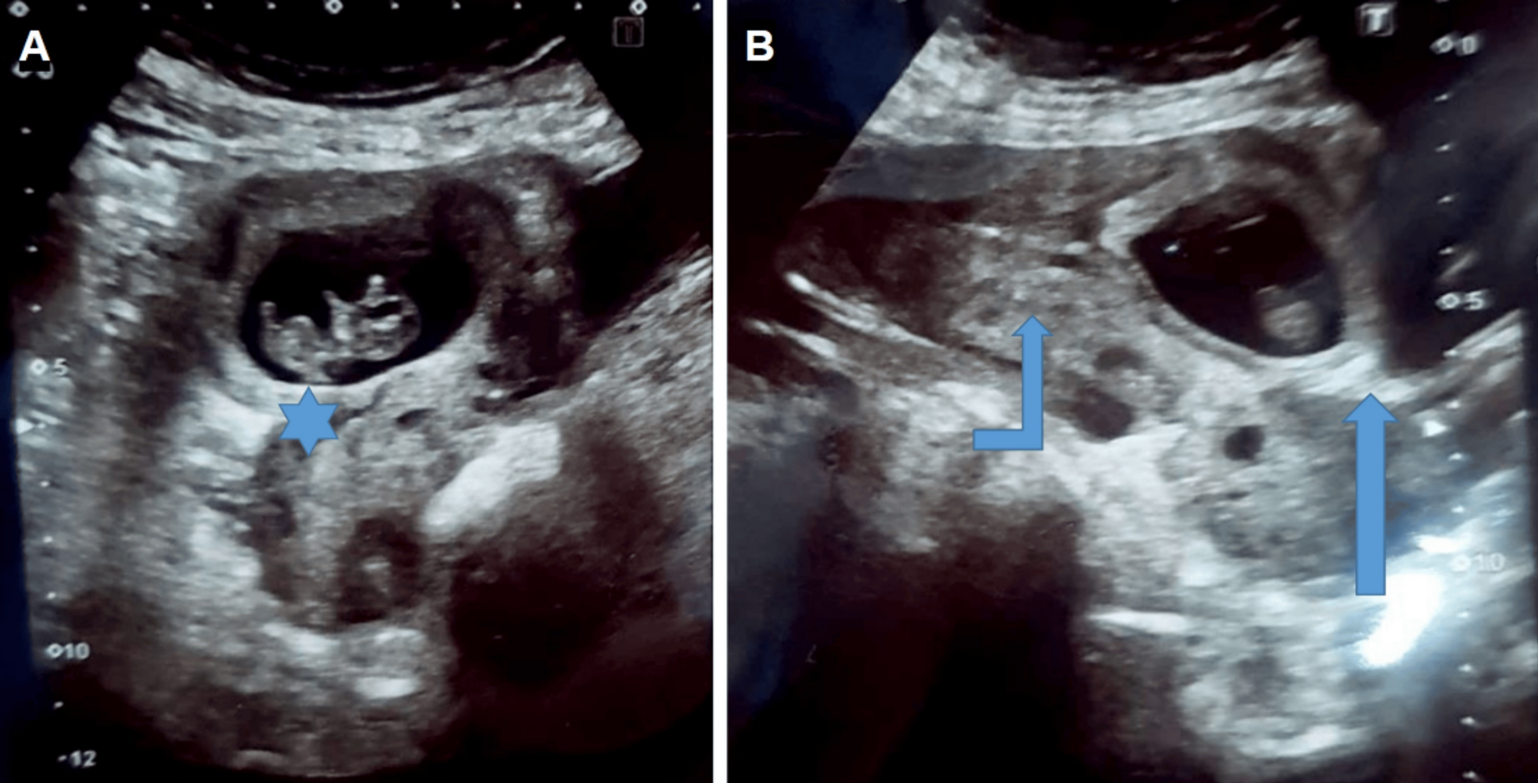
Transabdominal ultrasound pelvis A, B: Empty uterine cavity and cervical canal (curved arrow), gestational sac containing viable fetus (asterisk) in anterior lower uterine segment with no intervening myometrial tissue between bladder wall and uterus (straight arrow).

A 34-year-old woman with a history of cesarean delivery presented to an obstetric clinic with lower abdominal pain and nine weeks of amenorrhea. A urine pregnancy test was positive. A transabdominal pelvic ultrasound revealed an empty uterine cavity and an empty cervical canal with a gestational sac containing a viable fetus in the anterior lower uterine segment, compatible with a CSEP diagnosis. Importantly, there was no myometrial tissue observed between the bladder wall and the uterus, confirming the diagnosis (Figure 1). She underwent a successful surgical resection of the ectopic pregnancy with no immediate postoperative complications.

A transvaginal ultrasound was not used because the patients did not consent to this being done. The patient underwent laparotomy with scar excision. Since there is only one laparoscopic operating room accessible, which is mostly used for general surgery, laparoscopy is not frequently employed in our setting. As a result, all obstetrics and gynecological procedures are carried out using conventional open techniques.

## Discussion

CSEP is a rare but more frequently encountered form of ectopic pregnancy, with incidence estimates between one in 1,800 and one in 2,200 pregnancies. Depending on individual cases of ectopic pregnancies, the incidence has increased with more c-sections being performed [6]. Systematic reviews show that in the cases with fetal cardiac activity, expectant management results in the live birth of a child approximately 73% of the time but has a high risk (up to 70%) of uterine rupture or hemorrhage needing acute hysterectomy [7-9]. Therefore, expectant or conservative management is generally discouraged with active fetal heart activity.

CSEP treatment approaches are diverse and include medications (systemic or local methotrexate), minimally invasive surgical procedures, uterine artery embolization, suction aspiration, and open or laparoscopic surgical excision [6]. With methotrexate alone, the likelihood of success is only about 62% [8]. Suction curettage, particularly when performed in conjunction with uterine artery embolization, decreases the risk of bleeding complications. Comprehensive statistics suggest that surgical excision and repair of the scar defect had a high success rate (≥ 96%) and a low associated rate of bleeding complications (≤ 4%); they highlight the potency of surgical management as definitive treatment [8].

Recent case series from tertiary institutions support safe ultrasound-guided suction aspiration with minimal blood loss in pregnancies less than 50 days of gestation [10]. Embolization or other adjunctive methods may be needed to control bleeding in longer gestations (> 50 days). Although the present case involved surgical resection after about nine weeks of gestation (~63 days), the case was straightforward and exemplified the benefits of individualized, multidisciplinary planning [10].

After surgery, reproductive outcomes are promising. In a large retrospective review of 549 women who underwent uterine aspiration or laparoscopic scar repair, the overall live birth rate was 43%, with recurrent CSEP rates at 15% and secondary infertility at 38%. Notably, prior scar repair status did not influence rates of pregnancy [7-9,11-14]. In a multicenter cohort of 105 surgical patients, re-pregnancy was noted in 52%, the rate of recurrent CSEP was 13%, and the rates of live birth were equal between scar excision and no scar excision patients. In the cohort, scar excision during surgery was associated with significantly lower recurrence rates [15].

In the present case, the lack of uterine rupture or substantial bleeding and complete surgical removal of the product of conception without scar excision are also consistent with ideal surgical outcomes. Based on the evidence, uterine preservation and fertility are achievable outcomes with diligent treatment.

Limitations

This case does present some limitations. Namely, it is a single case, and a transvaginal ultrasound was not done, which is usually the main protocol for diagnosing similar cases. 

## Conclusions

CSEP is a rare disease that can have serious and often lethal consequences if it is not identified early. CSEP requires treatment in a timely fashion to prevent life-threatening complications such as uterine rupture and massive blood loss. The diagnosis is mostly based on imaging and requires providers to have a high index of suspicion for anyone with a history of cesarean delivery, regardless of gestation. Surgical excision is more invasive but has the potential for definitive treatment and future fertility preservation when done cautiously. Ultimately, all treatment is personalized, and consultation with a multidisciplinary team, which includes an obstetrician-gynecologist, risk management, and shared decision-making, can help prioritize maternal safety while still maximizing future fertility.
